# Risk factors for in-hospital mortality after coronary artery bypass grafting in patients 80 years old or older: a retrospective case-series study

**DOI:** 10.7717/peerj.2667

**Published:** 2016-12-01

**Authors:** Jacek Piątek, Anna Kędziora, Janusz Konstanty-Kalandyk, Grzegorz Kiełbasa, Marta Olszewska, Bryan HyoChan Song, Karol Wierzbicki, Irena Milaniak, Tomasz Darocha, Dorota Sobczyk, Bogusław Kapelak

**Affiliations:** 1 Institute of Cardiology, Jagiellonian University Medical College, Krakow, Poland; 2 Department of Cardiovascular Surgery and Transplantology, John Paul II Hospital, Krakow, Poland; 3 Cardiosurgical Students’ Scientific Group, Jagiellonian University Medical College, Krakow, Poland; 4 First Department of Cardiology, Interventional Electrocardiology and Hypertension, Jagiellonian University Medical College, Krakow, Poland; 5 Department of Anesthesiology and Intensive Care, John Paul II Hospital, Krakow, Poland; 6 Admission and Emergency Department, John Paul II Hospital, Krakow, Poland

**Keywords:** CABG, In-hospital mortality, Elderly patients, Postoperative complicatios, Risk factors

## Abstract

**Background:**

Age remains a significant and unmodifiable risk factor for cardiovascular diseases, and an increasing number of patients older than 80 years of age undergo Coronary Artery Bypass Grafting (CABG). Old age is also an independent risk factor for postoperative complications. The aim of this study is to describe the population of patients 80 years of age or older who underwent CABG procedure and to assess the mortality rate and risk factors for in-hospital mortality.

**Methods:**

A retrospective case-series study analyzing 388 consecutive patients aged 80 years of age or older who underwent isolated CABG procedure between 2010 and 2014 in the Department of Cardiovascular Surgery and Transplantology, John Paul II Hospital, Krakow.

**Results:**

In-hospital mortality stood at 7%, compared to 3.4% for all isolated CABG procedures at our Institution. In an univariate logistic regression analysis, risk factors for in-hospital mortality were as follows: NYHA class (*p* = 0.005, OR 1.95, 95% CI [1.23–3.1]), prolonged mechanical ventilation (*p* < 0.001, OR 7.08, 95% CI [2.47–20.3]), rethoracotomy (*p* = 0.04, OR 3.31, 95% CI [1.04–10.6]), duration of the procedure and ECC (for every 10 min *p* = 0.01, OR 1.01, 95% CI [1.0–1.01]; *p* = 0.03, OR 1.01, 95% CI [1.0–1.02], respectively), PRBC, FFP, and PLT transfusion (for every unit transfused *p* = 0.004, OR 1.42, 95% CI [1.12–1.8]; *p* = 0.002, OR 1.55, 95% CI [1.18–2.04]; *p* = 0.009, OR 1.93, 95% CI [1.18–3.14], respectively). Higher LVEF (*p* = 0.02, OR 0.97, 95% CI [0.94–0.99]) and LIMA graft implantation (*p* = 0.04, OR 0.36, 95% CI [0.13–0.98) decreased the in-hospital mortality. Death before discharge was more often observed in patients with multiple risk factors for cardiovascular diseases (0–2 –5.7%; 3–7.4%, 4–26.6%; *p* = 0.03).

**Conclusions:**

Older age is associated with higher in-hospital mortality after isolated CABG at our Institution. Risk stratification scores and individualized risk evaluation, centered on comorbidities, NYHA class and left ventricular function, should be assessed in all cases. Whenever suitable, LIMA grafts should be used. Prolonged procedure and ECC time worsen the short-term outcome. Elderly individuals should be closely monitored postoperatively and the care should be focused on excessive blood loss and respiratory failure.

## Introduction

Civilization development has prolonged longevity in developed countries, and statistics show that 6.2% of the American society has reached the age of 80 years old. The expected life time for this population reached 8.1 years (United Nations, Department of Economic, Social Affairs Population Division, 2013). Age remains a significant and unmodifiable risk factor for cardiovascular diseases and nowadays, more than 20% and 30% of octogenarians and nonagenarians have vascular disease in at least one arterial territory (Savji et al., 2013). Therefore, an increasing number of patients older than 80 years old undergo Coronary Artery Bypass Grafting (CABG), reaching 7.3% of all CABG procedures at our Institution. Older age is also an independent risk factor for postoperative complications (Weisel et al., 2014).

Currently, mortality rates for patients over 80 years old are higher than those observed for younger groups. Studies show that the 30-day mortality and the incidence of postoperative complications increase significantly with age. In the analysis of 6,057 patients who underwent isolated CABG between 1996 and 2002, the 30-day mortality rate and the incidence of postoperative complications were found to largely escalate with age (Mortasawi et al., 2004). In-hospital cost and outcomes were examined among 2,272 elderly patients (≥75 years old) and 9,745 younger patients (<75 years old) who underwent CABG between 1997 and 2001 in another study with similar results (Chee et al., 2004). These referenced studies showed that the cost of hospitalization in older patients who undergo CABG are greater. The observed differences are mostly accounted by the extended hospital stay and higher incidence of postoperative complications (i.e., pneumonia, deep wound infections) (O’Neill et al., 2016; Toor et al., 2009).

The risk stratification scores which are the most commonly used, Euroscore II and STS risk calculator, include age as a part of preoperative risk, but there is a gap in knowledge regarding risk factors for poor outcomes in the age group of 80 years old and older patients. As the risk of surgery is usually high in elderly, studies focused on defining geriatric-specific risk are warrantable.

The aim of this study is to describe the population of patients 80 years old or older who underwent CABG procedure and to assess the mortality rate and risk factors for in-hospital mortality.

## Methods

In a retrospective case-series study, we analyzed 388 consecutive patients aged 80 years old or older who underwent isolated CABG procedure between 2010 and 2014 in the Department of Cardiovascular Surgery and Transplantology, John Paul II Hospital, Krakow. Data was obtained from patient medical records. All patients in the analysis were qualified for surgical revascularization according to European Society of Cardiology (ESC) guidelines (Windecker et al., 2014).

Preoperative evaluation including baseline demographic data (age, sex, weight, BMI), the prevalence of risk factors for CAD and other comorbidities (diabetes, hypertension, hyperlipidemia, history of previous cardiovascular events—MI, stroke, PCI, CABG, atrial fibrillation (AF), chronic kidney disease (CKD), chronic obstructive pulmonary disease (COPD), peripheral artery disease (PAD)) was performed for all patients. Patients with BMI between 25 and 30 kg/m^2^ were classified overweight, while ≥30 kg/m^2^ were classified obese, in congruence with the WHO classificiation. Left ventricular ejection fraction (LVEF), current CCS and NYHA class were also assessed on admission. Mode of the admission (emergent or elective) was also included in the analysis.

CABG On-Pump procedures were performed with placement of arterial grafts (left internal mammary artery (LIMA), right internal mammary artery (RIMA), and radial artery (RA)), venous grafts (saphenous vein graft (SVG)), or both. Arterial and venous grafts were used based on indications covered by our institution’s protocol and surgeon’s preferences. General anesthesia was carried out using propofol as a hypnotic, sufentanyl as an analgesic, and non-depolarizing muscle relaxant. Heparinization was achieved before starting a Cardiopulmonary Bypass (CPB) and subsequently reversed with protamine. Our institution’s standard protocol includes heparin 3 mg/kg and protamine 1:1. Blood or crystalloid cardioplegia was used for all cases. Distal and proximal anastomoses were attached with continuous running sutures, using non-absorbable Prolene^®^.

When suitable, Off-Pump surgery was performed. Preoperative care and anesthesia were carried out following same protocols. Distal anastomoses were performed as during On-Pump procedure with or without tissue stabilizers.

All surgeries were performed via median sternotomy.

### Statistical analysis

Statistical analysis was performed using STATISTICA software, version 10.0. In order to confirm a normal distribution of continuous variables, the Shapiro–Wilk test was used. Results were presented based on the parameters of descriptive statistics, including mean values and its standard deviations, or median values and its quartiles, as appropriate. Categorical variables were presented as percentages. Continuous variables were compared via Student’s *t*-test and categorical variables via Chi-square test. Univariate and multivariate stepwise logistic regression wereused to determine risk factors for in-hospital mortality. A *p* value less than 0.05 was considered significant.

The study was approved by the Chief of the Department of Cardiovascular Surgery and Transplantology, John Paul II Hospital, Kraków. Based on Medical Profession Act (Dz.U. 1997 nr 28 poz. 152) and the Polish Code of Medical Ethics, a retrospective analysis of patient medical documentation needs no further evaluation by external or internal review boards. All patients consented to the study in writing.

## Results

Analyzed population comprised mostly of overweight (68%) males (68%) with a mean age of 82.4 ± 2.3 years old. The preoperative risk factors included accompanying hypertension in 85.8% of individuals, diabetes in 32.7% diabetes, and hyperlipidemia 13.9%. Most patients had history of myocardial infarction (MI) (63.4%), but minority of the study cohort had been previously treated with Percutaneous Coronary Intervention (PCI) (18.8%). Among the study population, 13.9% of the individuals suffered from atrial fibrillation, 5.9% from Chronic Obstructive Pulmonary Disease (COPD), and 19.6% had Chronic Kidney Disease (CKD). Most of the patients had three-vessel disease (62.6%) with affected left main (LM) (46.1%). Mean Left Ventricle Ejection Fraction (LVEF) was 48 ± 13% (Table 1).

**Table 1:** Baseline characteristics.

Variables	Analyzed population, *n* = 388
Age, years	82.4 (±2.3)
Male sex, *n* (%)	264 (68)
BMI, kg/m^2^	27.12 (±3.8)
Normal weight, *n* (%)	119 (30.7)
Underweight, *n* (%)	5 (1.3)
Overweight, *n* (%)	179 (46.1)
Obese, *n* (%)	85 (21.9)
Diabetes, *n* (%)	127 (32.7)
Hypertension, *n* (%)	333 (85.8)
Hyperlipidemia, *n* (%)	54 (13.9)
0 risk factors for CAD, *n* (%)	22 (5.7)
1 risk factors for CAD, *n* (%)	92 (23.7)
2 risk factors for CAD, *n* (%)	151 (38.9)
3 risk factors for CAD, *n* (%)	108 (27.8)
4 risk factors for CAD, *n* (%)	15 (3.9)
Atrial fibrillation, *n* (%)	54 (13.9)
COPD, *n* (%)	23 (5.7)
CKD, *n* (%)	76 (19.6)
CCS class 0, *n* (%)	5 (1.3)
CCS class I, *n* (%)	39 (10.1)
CCS class II, *n* (%)	98 (25.26)
CCS class III, *n* (%)	172 (44.3)
CCS class IV, *n* (%)	74 (19.1)
Single-vessel disease, *n* (%)	72 (18.6)
Two-vessel disease, *n* (%)	69 (17.8)
Three-vessel disease, *n* (%)	243 (62.6)
LM disease, *n* (%)	179(62.6)
LVEF, %	48 (±13)
Previous MI, *n* (%)	246 (63.4)
Previous PCI, *n* (%)	73 (18.8)

**Notes.**

Data shown as mean ± SD or as median (IQR), or number (percentage).

BMI Body Mass Index CAD Coronary Artery Disease COPD Chronic Obstructive Pulmonary Disease CKD Chronic Kidney Disease CCS Canadian Cardiovascular Society LM Left Main LVEF Left Ventricle Ejection Fraction MI Myocardial Infarction PCI Percutaneous Coronary Intervention

Most of the patients required two or more bypass grafts. Asides from LIMA, arterial grafts were not commonly used. Median procedure time, Aortic Cross-Clamp time, and Extracorporeal Circulation (ECC) time were 220, 40, and 82 min respectively. Emergent surgery was performed in 31.2% cases (Table 2).

In-hospital mortality was 7% (27 cases), and was higher than the mortality reported by the National Registry of Cardiosurgical Procedures in 2015 for all CABG procedures performed at our Institution (3.4%). All postoperative complications are presented in Table 3.

**Figure 1:**
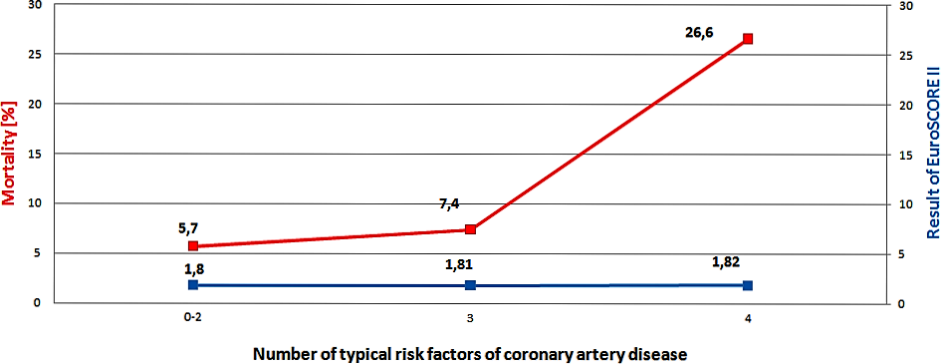
Relation between results of EuroSCORE II, mortality and number of typical risk factors of coronary artery disease. Out of BMI ≥ 25 kg/m^2^, hypertension, hyperlipidemia, diabetes.

None of the baseline risk factors for Coronary Artery Disease (CAD), including BMI > 25 kg/m^2^, hypertension, diabetes, and hyperlipidemia, influenced the risk of in-hospital mortality separately. However, death before discharge was more often observed in patients with multiple risk factors, out of BMI > 25 kg/m^2^, hypertension, diabetes, and hyperlipidemia, (0–2–5.7%; 3–7.4%, 4–26.6%; *p* = 0.03; median EuroSCORE II 0–2—1.80%; 3—1.81%; 4—1.82%; median STS score 4–1.5%) (Fig. 1).

**Table 2:** Intraoperative data.

Variables	Analyzed population, *n* = 388
Elective surgery, *n* (%)	267 (68.8)
Emergent surgery, *n* (%)	121 (31.2)
On-Pump surgery, *n* (%)	368 (94.85)
Off-Pump surgery, *n* (%)	20 (5.15)
Surgery time, minutes	220 (160–245)
Aortic Cross-Clamp time, minutes	40 (30–60)
Extracorporeal Circulation time, minutes	82 (65–100)
1 bypass graft, *n* (%)	70 (18)
2 bypass grafts, *n* (%)	203 (52.3)
3 bypass grafts, *n* (%)	104 (26.8)
4 bypass grafts, *n* (%)	11 (2.8)
LIMA graft, *n* (%)	144 (37.1)
SVG graft, *n* (%)	345 (88.9)
RIMA graft, *n* (%)	3 (0.8)
RA graft, *n* (%)	1 (0.3)

**Notes.**

Data shown as mean ± SD or as median (IQR), or number (percentage).

LIMA Left Internal Mammary Artery SVG Saphenous Vein Graft RIMA Right Internal Mammary Artery RA Radial Artery

**Table 3:** Postoperative complications.

Variables	Analyzed population, *n* = 388
Death, *n* (%)	27 (6.96)
MI, *n* (%)	4 (1.03)
Neurological complications, *n* (%)	4 (1.03)
MODS, *n* (%)	0 (0)
Respiratory complications, *n* (%)	20 (5.15)
Rethoracotomy, *n* (%)	22 (5.7)
IABP, *n* (%)	11 (2.8)
Sternal dehiscence, *n* (%)	15 (3.9)
AKI, *n* (%)	4 (1.03)
GI bleeding, *n* (%)	6 (1.55)
PRBC transfusions, *n* (%)	319 (82.2)
FFP transfusions, *n* (%)	140 (36.1)
PLT transfusions, *n* (%)	113 (29.1)

**Notes.**

Data shown as number (percentage).

MI Myocardial Infarction MODS Multiple Organ Dysfunction Syndrome IABP Intra-aortic Balloon Pump AKI Acute Kidney Injury GI Gastrointestinal PRBC Packed Red Blood Cells FFP Fresh Frozen Plasma PLT Platelets

**Figure 2:**
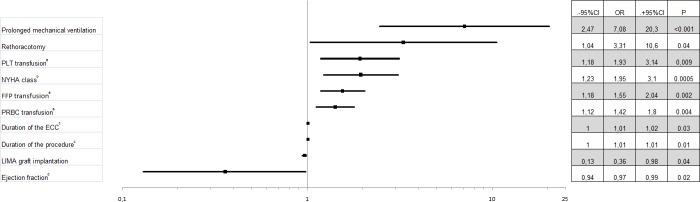
Factors influencing on in-hospital mortality. a—for every next transfused unit, b—for next higher class, c—for every next 10 min, d—for every 1% increase in left ventricular ejection fraction. Abbreviations: PLT, platelet concentrate; NYHA, New York Heart Association; FFP, fresh frozen plasma; PRBC, packed red blood cells; ECC, extracorporeal circulation; LIMA, left internal mammary artery.

Univariate logistic regression showed risk factors for in-hospital mortality: prolonged mechanical ventilation (*p* < 0.001, OR 7.08, 95% CI [2.47–20.3]), rethoracotomy (*p* = 0.04, OR 3.31, 95% CI [1.04–10.6]), duration of the procedure and ECC (for every 10 min *p* = 0.01, OR 1.01, 95% CI [1.0–1.01]; *p* = 0.03, OR 1.01, 95% CI [1.0–1.02], respectively), PRBC, FFP, and PLT transfusion (for every unit transfused *p* = 0.004, OR 1.42, 95% CI [1.12–1.8]; *p* = 0.002, OR 1.55, 95% CI [1.18–2.04]; *p* = 0.009, OR 1.93, 95% CI [1.18–3.14], NYHA class (*p* = 0.005, OR 1.95, 95% CI [1.23–3.1], respectively)). LIMA graft implantation and higher LVEF was observed to decrease the in-hospital mortality (*p* = 0.04, OR 0.36, 95% CI [0.13–0.98]; *p* = 0.02, OR 0.97, 95% CI [0.94–0.99], respectively) (Fig. 2).

Lastly, multivariate stepwise analysis showed that only NYHA class and PRBC transfusion were significant risk factors (*p* = 0.002, OR = 1.4 CI [1.13–1.75]; *p* = 0.003, OR = 2.05 CI [1.27–3.32], respectively). However, the predictive value of this model was low (AUC = 0.73 ± 0.06, result of Hosmer-Lemeshow test = 8.04, *p* = 0.15).

## Discussion

Age is a significant risk factor for poor outcome after cardiac procedures and is included in all risk stratification scores. Similar high in-hospital mortality was observed in the study cohort (7% vs 3.4% for all isolated CABG at our Institution). Furthermore, comorbidities in elderly patients are described to be associated with negative outcomes (Weisel et al., 2014; Higgins et al., 1992). In the presented study, the presence of multiple comorbidities significantly increased the risk of in-hospital mortality, reaching 26.7% for patients with all 4, out of BMI > 25 kg/m^2^, hypertension, hyperlipidemia, and diabetes. This mortality rate was higher than it was expected after assessing Euroscore II and STS score. Thus, the presented results suggest that the current standard risk stratifications may not suffice for older patients as it is for the younger age groups.

Moreover, higher NYHA class was associated with increased in-hospital mortality and the aggravation of heart failure symptoms should be carefully evaluated while assessing the procedure risk for elderly patients. On the other hand, individuals with preserved left ventricular function, defined by the preoperative LVEF, had better survival rates.

The study cohort required emergent CABG more often than that of younger age groups. A total of 31.2% of the cases were performed in the presence of Acute Coronary Syndrome (ACS), compared to 18.9% observed for all age groups at our Institution. The emergency of the procedure is described in the literature as a significant risk factor for poor outcome after cardiac surgeries (Creswell et al., 1995; Kurki, Kataja & Reich, 2003). However, emergent surgery was not associated with higher rates of in-hospital mortality in the study group. This may be due to the fact that ACS in elderly patients may be less damaging, because of the collateral blood flow that had developed over time. Moreover, acute plaque ruptures and complex lesions, that are associated with worse outcome after ACS, are observed less frequently in older age groups (Zimmerman et al., 1995; Kusama et al., 2007). Therefore, in spite of the presence of critical stenosis in the coronary artery that require emergent revascularization, the outcome of the procedure may not be influenced by the high surgery priority. To our best knowledge, the influence of performing an emergent CABG procedure on the in-hospital mortality has not been previously analyzed between different age groups. Results from the study cohort suggest that there is no need in delaying the procedure in patients ≥80 years old.

Although the LIMA graft is a gold-standard option for LAD occlusions, it was used in less than half of the cases. However, implanting a LIMA graft was associated with lower in-hospital mortality. Similar observations are made by other researchers. LIMA use was found to be one of the highest predictive factors for 30-day and 180-day survival in septuagenarians, and have been reported similarly for other age groups (OR 0.480 for 30-day and OR 0.529 for 180-day mortality) (Arif et al., 2016; Leavitt et al., 2001). When LIMA grafts were first introduced in 1985, the mortality rate for elderly patients stood at 9.3% and has fallen since then to 5.5%. Major surgical complications have been either reduced or unchanged in patients receiving LIMA grafts (Gardner et al., 1990). However, LIMA grafts have not yet become the standard protocol for elderly patients in our study cohort presumably due to relative contraindications, which include left ventricular hypertrophy, severe left ventricular dysfunction, emergency operations, COPD with enlarged lungs, advanced age, and an obstructed left subclavian artery (Leavitt et al., 2001; Gardner et al., 1990). Another reason may be that more of the coronary angiograms are nowadays performed via radial access and this results in the difficulty of obtaining the LIMA angiogram. Some surgeons may then withdraw from harvesting LIMA. However, the benefits of LIMA graft implantations outweigh the contraindications and should have been performed in more cases. Lastly, early results from our institution show that there is no difference in postoperative sternal dehiscence occurrence, when a single or both internal mammary arteries were harvested in the elderly, compared to venous grafts.

Duration of the procedure and ECC were determined to be significant risk factors for in-hospital mortality in the study cohort, with the increase for every 10 min. Similar results for surgical procedures, other than cardiac, have been previously described and a 30-min increase in procedure duration resulted in 17% higher odds of mortality in patients older than 80 years old (Turrentine et al., 2006). As the risk increased significantly for every 10 min of the procedure and ECC, the range of the procedure and the number of necessary bypass grafts should be closely discussed with a Heart Team preoperatively. Moreover, prolonged ECC time may contribute to hemolysis, blood loss, and need for blood products transfusions, that were also associated with higher in-hospital mortality in the study cohort. Therefore, the impact of these complications on the short-term outcome may be synergic. Similar results, concerning the influence of excessive blood loss have been previously described for general population of patients undergoing cardiac surgeries (Diaz-Martin et al., 2010; Kinduris, Vaisvila & Budrikis, 2006). Moreover, re-exploration for bleeding is commonly concerned as an independent risk factor for prolonged hospitalization and mortality in cardiac surgery and more deaths before discharge were also observed in patients, who required this procedure (Mehta et al., 2009).

Furthermore, prolonged mechanical ventilation resulted in the increased mortality rate. This association is observed after cardiac procedures for all age groups, but the high OR value suggests relatively strong dependence in elderly patients. The risk of postoperative respiratory insufficiency have been observed to increase with age and have been associated with worse outcome for all groups (Starr & Stefan, 2016; Gumus et al., 2015). It negatively influences postoperative pulmonary rehabilitation, and increase the risk of infection. The association is most likely higher in elderly patients due to the lower lung compliance. Normal aging of the pulmonary system decreases overall pulmonary reserve. As a result, older adults are more susceptible to respiratory compromise in the postoperative period and during monitored anesthesia care (Sprung, Gajic & Warner, 2006). Moreover, in a systematic review prepared for the American College of Physicians, in patients aged ≥80 years old OR of postoperative pulmonary complications stood at 5.63 (95% CI [4.63–6.85]) compared to patients <50 years old (Smetana et al., 2006).

Lastly, in-hospital mortality observed in the study cohort was higher than that of the general population. The results indicate that one of the factors contributing might result from rare implantation of LIMA graft, which provides better blood supply to the anterior heart wall, compared to venous grafts. Moreover, multiple comorbidities were associated with high risk of death before discharge. Although the risk score stratifications were high in the subgroup of 4 or more comorbidities, the actual mortality was higher than expected, with longer duration of the procedure and ECC contributing. Because of this high rate of mortality, elderly patients with multiple comorbidities should be considered as candidates for percutaneous or hybrid procedures, and their cases should be closely discussed with the Heart Team. It is further significant to note that the benefit of CABG in the elderly observed at six months did not persist at 12 months observation (Zhang et al., 2006). Lastly, in case of a CABG procedure, LIMA grafts should be implanted, whenever possible. A subset of patients with suitable anatomy may benefit from a minimally-invasive off-pump CABG or MIDCAB of LIMA to LAD combined with percutaneous coronary intervention to other significantly diseased vessels. This approach may be particularly beneficial in patients with contraindications for CPB (atherosclerotic aorta, chronic obstructive pulmonary disease, renal dysfunction). However, a large randomized control trial comparing hybrid revascularization and conventional CABG, with mid-term follow-up, is required to establish the clinical effectiveness  (Jones, Qiu & Sudarshan, 2010).

The greatest study limitation is a retrospective design that does not allow to draw any definite conclusions concerning managing revascularization in patients ≥80 years old. However, the univariate logistic regression analysis identified risk factors for in-hospital mortality in the study cohort. The stepwise analysis excluded most of the variables, but the predictive value of this model was low, presumably due to the high clinical association between tested variables.

## Conclusions

Higher in-hospital mortality is observed in older patients after isolated CABG at our Institution. This study demonstrated that a greater number of preoperative risk factors (from BMI ≥ 25 kg/m^2^, hypertension, hyperlipidemia, diabetes), lower LVEF, and higher NYHA class increases the in-hospital mortality in patients ≥80 years old. The observed mortality in all groups was higher than that predicted by the EuroSCORE II. Notably, the difference in mortality rate became greater as the number of risk factors increased. Among elder patients, the LIMA graft should be used whenever suitable as it is associated with better survival after hospital discharge. Prolonged procedure and ECC time worsened the short-term outcome in the study cohort. Therefore, hybrid procedures should be considered for patients ≥80 years old. Elderly individuals who required blood product transfusions or rethoracotomy, and needed prolonged mechanical ventilation, showed higher in-hospital mortality and should be considered at higher risk for death before discharge. Elderly patients should be carefully discussed with the Heart Team to ensure the best treatment option.

##  Supplemental Information

10.7717/peerj.2667/supp-1 Data S1 Raw data exported from patient medical histories used for data analyses

## Additional Information and Declarations

### Data Availability

The following information was supplied regarding data availability: The raw data has been supplied as Data S1.
